# Precision postoperative pain management: A multidimensional matter

**DOI:** 10.1177/17504589251391942

**Published:** 2025-12-08

**Authors:** Timothy Trewren, Timothy Falloon, Samuel West, Brandon Stretton, Joshua Kovoor, Aashray Gupta, Stephen Bacchi

**Affiliations:** 1 Adelaide Medical School, The University of Adelaide, Adelaide, SA, Australia; 2 Flinders University, Bedford Park, SA, Australia; 3 Gold Coast University Hospital, Southport, QLD, Australia; 4 Lyell McEwin Hospital, Elizabeth Vale, SA, Australia; 5 Harvard Medical School, Boston, MA, USA

**Keywords:** Postoperative pain management, Multimodal analgesia, Opioid-sparing strategies, Precision medicine, Individualised care

The effective management of acute postoperative pain has long challenged health care practitioners. Pain is now recognised as a multidimensional experience involving biological, psychological, and social factors (Schreiber et al 2019). Traditionally, postoperative pain was solely attributed to a nociceptive response to tissue injury; however, contemporary research demonstrates a more complex interplay of factors which includes peripheral and central sensitisation, as well as the patient’s mind-set towards pain, as significant contributors to the overall patient experience (Pak et al 2018). Despite vast improvements in our understanding of pain and greater investment in acute pain services available to patients, recent data have shown a significant proportion of patients still experience undesirable levels of postoperative pain. A subgroup analysis of prospective and controlled data from adult patients who underwent short-stay surgery demonstrated severe dynamic pain in half of patients in the recovery unit despite the majority receiving multimodal analgesia (Lappalainen et al 2022). This viewpoint article aims to highlight the challenges clinicians face in managing acute postoperative pain and to advise on key aspects that should be recognised to improve patient outcomes through a more individualised approach.

The first step in conceptualising postoperative pain is to understand the factors which predispose patients to higher levels of pain and then consider the subsequent complications that may arise from postoperative pain. A study of risk factors leading to inadequate pain control postoperatively identified anxiety and other psychological conditions, current opioid use, and current smoking as the most significant indicators of higher postoperative pain scores (Armstrong et al 2020). Failing to identify these patients will lead to a greater burden of pain and subsequently trigger a cascade of problematic physiological and pathological adaptations. An observational study of 1014 surgical patients demonstrated an increased incidence of nausea, vomiting, ileus, gastroparesis, constipation, atelectasis, and health care–associated infections with higher postoperative pain scores (Van Boekel et al 2019). Postoperative pain impairs mobility and coughing, which may lead to atelectasis and increased risk of respiratory infection. Gastrointestinal dysfunction after surgery can result from both postoperative pain and the side effects of opioids. The same pattern is seen in the immune system, where poor pain control and the impact of opioid therapy each contribute to postoperative immunosuppression (Zajączkowska et al 2018). Pain and physiological stress activate pathways that drive the autonomic stress response, protein breakdown, and the release of cortisol, catecholamines, and glucagon (Soop & Ljungqvist 2025). Together with other postoperative changes, these responses further suppress the immune system (O’Dwyer et al 2015) and may increase the risk of health care–associated complications.

These factors may be compounded in racially and culturally heterogeneous communities due to variability in the delivery of postoperative pain relief. There is evidence that ethnicity-based differences in pain care exist across all settings where pain relief is administered (Thompson et al 2024), leading to racial minorities being provided with suboptimal analgesia when compared to Caucasian patients. Language barriers may also factor into this, with one study demonstrating that children with parents who had a limited English proficiency received fewer pain assessments and were less likely to receive opioid analgesia (Jimenez et al 2014). It is clear that education for health care providers regarding social and cultural factors, and increasing utilisation of services such as professional translators, are essential components of improving postoperative pain management.

The current standard of postoperative pain management consists of a holistic approach including pharmacological and non-pharmacological methods. Pharmacotherapy for acute pain is focused on the delivery of multimodal analgesia with the use of opioid-sparing techniques where possible. This typically involves administration of paracetamol, non-steroidal anti-inflammatory drugs, ketamine and gabapentinoids as an adjunct to opioids, alongside regional anaesthesia (Wick et al 2017). Non-pharmacological interventions to aid the management of postoperative pain include transcutaneous electrical nerve stimulation, acupuncture, massage, cold therapy, and cognitive behavioural modalities; however, there is limited evidence indicating the efficacy of these methods across all patient populations (Chou et al 2016).

Opioid therapy remains as the foundation of postoperative pain management, providing highly effective relief for moderate-to-severe pain (Rawal 2016); however, this is not without downsides. Opioids are associated with an extensive array of dose-limiting adverse effects including nausea, constipation, sedation, and respiratory depression, all of which contribute to an increased cost and length of hospital stay (Oderda et al 2013). Furthermore, the use of opioids in the acute pain setting poses an inherent risk of dependence and addiction which has been escalating within the general population and highlights the importance of judicious use (Hah et al 2017). The application of multimodal analgesia in surgical departments to prevent opioid dependence and sensitisation has also been demonstrated to be suboptimal, with opioids remaining as a first-line therapy with inadequate non-opioid analgesia (Mathiesen et al 2012). Furthermore, there is a growing body of evidence that the backbone of multimodal analgesia should include regional techniques, in particular surgeon-administered infiltrative techniques involving local anaesthesia (Ray et al 2022). Multimodal analgesic techniques have also shown improvements in the support of good immune function postoperatively (Zong et al 2021).

It is likely there will continue to be a paradigm shift away from the ‘one-size-fits-all’ approach of yesteryear, with procedure-specific guidelines for pain management facilitating optimal pain control for all patients. It is clear that postoperative pain management is trending towards varying methods of avoiding overutilisation of opioids, often with a combination of approaches to provide synergistic benefit to the pain experience of the patient. This positive shift towards individualised, multimodal strategies marks a pivotal step in optimising postoperative pain control.
